# Ethical Considerations in Chemotherapy and Vaccines in Cancer Patients in Times of the COVID-19 Pandemic

**DOI:** 10.3390/curroncol28030186

**Published:** 2021-05-26

**Authors:** Guido V. Schiappacasse

**Affiliations:** 1 Oncology Department, Clinical Hospital of Viña del Mar, Limache Street 1741, Viña del Mar 2520000, Chile; g.schiapp@hotmail.com; Tel.: +56-959021201; 2 Oncology Department, Bupa Reñaca Clinic, Anabaena Street 336, Viña del Mar 2520000, Chile

**Keywords:** COVID-19, cancer, chemotherapy, vaccines, medical ethics

## Abstract

The COVID-19 situation is a worldwide health emergency with strong implications in clinical oncology. In this viewpoint, we address two crucial dilemmas from the ethical dimension: (1) Is it ethical to postpone or suspend cancer treatments which offer a statistically significant benefit in quality of life and survival in cancer patients during this time of pandemic?; (2) Should we vaccinate cancer patients against COVID-19 if scientific studies have not included this subgroup of patients? Regarding the first question, the best available evidence applied to the ethical principles of Beauchamp and Childress shows that treatments (such as chemotherapy) with clinical benefit are fair and beneficial. Indeed, the suspension or delay of such treatments should be considered malefic. Regarding the second question, applying the doctrine of double-effect, we show that the potential beneficial effect of vaccines in the population with cancer (or those one that has had cancer) is much higher than the potential adverse effects of these vaccines. In addition, there is no better and less harmful known solution.

## 1. Introduction

One year into the COVID-19 pandemic, the outlook is disheartening. The World Health Organization reported, on 27 February 2021, almost 113 million confirmed cases worldwide, with more than 2.5 million deaths (global mortality rate of 2.2 per 100 inhabitants). In this regard, the Americas and Europe are the regions with the highest number of infections, and the United States has had more than 28 million confirmed cases with more than 500 thousand deaths from this cause. Consequently, this global infection has had a strong impact regarding decision-making in clinical oncology. It is particularly important to determine if decision-making in oncology clinical is ethical in the COVID-19 context.

In this viewpoint, we address two crucial dilemmas from the ethical dimension: (1) Is it ethical to postpone or suspend cancer treatments which offer a statistically significant benefit in the quality of life and survival in cancer patients during this time of pandemic?; (2) Should we vaccinate cancer patients against COVID-19 if scientific studies have not included this subgroup of patients?

Regarding the first question, the best available evidence shows that:

(a) A systemic review (*n* = 52 studies with 18,650 cases) revealed that patients with cancer and COVID-19 infection had a higher mortality than the general infected population without cancer (mortality of 25.6 per 100 inhabitants). Cancer is an independent risk factor for mortality (especially hematologic malignancies and lung cancer). Other risk factors are age, the male gender, the black race, current smoking habit, and comorbidities [1];
(b) A single-center, retrospective, observational study (*n* = 309 cases) showed that in patients with cancer who are COVID-19-positive, cytotoxic chemotherapy administered within 5 weeks of the diagnosis of viral infection does not significantly increase the rate of events which are serious or critical for COVID-19. In contrast, the presence of hematological neoplasms, lung cancer, lymphopenia at the time of diagnosis of COVID-19 infection, and initial neutropenia (14 to 90 days prior to diagnosis of this viral infection) are significantly associated with a higher rate of critical or serious events due to this infection [2];
(c) A prospective observational study (*n* = 800 cases) showed that in cancer patients who test positive for COVID-19 (through RT-PCR in nose or throat smears), after adjusting for other risk factors, patients who underwent cytotoxic chemotherapy showed no significant effect on mortality versus cancer and COVID-19-positive patients who did not receive chemotherapy. It should be noted that neither adjuvant chemotherapy nor palliative chemotherapy showed any significantly higher mortality within 4 weeks of the infection diagnosis versus the group that received no treatment. When comparing recent first-line palliative chemotherapy versus second- or third-line chemotherapy, mortality did not vary significantly in cancer patients who were COVID-19-positive. There was also no significant difference in mortality with respect to immunological therapy, hormonal therapy, radiotherapy, and target therapies between the group that received early treatment versus those who did not [3];
(d) The suspension or delay in cancer therapy during this pandemic, based on a supposed greater possibility of harm to the patient, is a reality. For example, in the previous study, treatment was interrupted due to the current pandemic situation in 22% of patients (*n* = 172 cases) [3]. In a prospective study in COVID-19-negative lung cancer patients (*n* = 211 cases), it was determined that palliative chemotherapy was delayed in 39.7% of cases due to the pandemic, was interrupted in 14.9%, and was definitively stopped in 3% [4,5].

Regarding the second question, the epidemiological course of this infection shows that hygienic and isolation measures, although necessary, are not sufficient to control the epidemic. Mass immunization measures are required. Therein, the FDA hastened the approval of messenger RNA vaccines (BNT162b2 and mRNA-1273) and the Ad26.COV2.SCOVID-19 vaccine (recombinant replication-incompetent adenovirus serotype Ad26 vector encoding a full-length and stabilized SARS-Cov-2 spike protein).

From the analysis of the best available evidence, the BNT162b2 vaccine shows 95% efficacy in preventing symptomatic infection. Furthermore, efficacy is similar in subgroup analyses defined by age, sex, race, ethnicity, baseline body mass index, and comorbidities; the safety of this vaccine is similar to other viral vaccines [6].

The mRNA-1273 vaccine is 94.1% effective in preventing symptomatic infection. This efficacy was consistent across all stratified subgroups (by age, sex, race, ethnicity, and high-risk groups for severe infection) and presented acceptable level of safety [7].

The Ad26.COV2.SCOVID-19 vaccine shows high level of safety and reactogenicity in healthy cases of different ages [8]. The interim analysis assessed 468 cases of symptomatic COVID-19 among 44,325 adult volunteers in South America, South Africa and the United States. The investigational vaccine was reportedly 66% effective at preventing the study’s combined endpoints of moderate and severe COVID-19 at 28 days post-vaccination among all volunteers, including those infected with an emerging viral variant (press release, 29 January 2021).

In this regard, the American Cancer Society recommends that patients with (or who have had) cancer receive the vaccine as soon as it becomes available. However, the studies do not include subgroups with cancer patients. Therefore, it is worth considering whether it is ethical to administer this therapy in cancer patients, without clear scientific evidence on the efficacy and adverse effects of these vaccines in this group of patients.

## 2. Comment Development

### 2.1. First Bullet

Regarding the first question, we present the following analysis:

First item

The ethics of Beauchamp and Childress postulate four basic principles to know: autonomy, beneficence, non-maleficence, and justice [9].

That said, considering Diego Garcia’s hierarchy of ethical principles, we have the ethics of minimums (accounting for the public dimension of humans in their relationships with others, required in all circumstances), which encompasses the principle of non-maleficence and justice. At a lower level, we find that the ethics of maximums (which account for the private sphere, not required at all times) and its principles are beneficence and autonomy [10].

If we apply these principles, starting from the higher (principles of the ethics of minimums) to the subordinate level (principles of the ethics of maximums), and consider the problem at hand, it follows that:

(a) Non-maleficence: understood as not causing harm voluntarily. Evidence shows that when chemotherapy is administered early in cancer patients who are COVID-19-positive, this does not increase the risk of severe morbidity from this infection, nor does it increase mortality. Therefore, it is malicious to delay adjuvant, neoadjuvant, or palliative treatment that has been shown to improve the quality of life and survival in cancer patients. We only increase the risk of increasing morbidity and mortality from the underlying neoplastic disease, managing to do harm by omission. It is understood that we are referring to treatments which have demonstrated clinical benefit. On the other hand, if, and only if, the chemotherapy in question does not improve the patient’s quality of life and survival with certainty versus placebo, then the suspension of the therapy would be legitimate. It must be emphasized that at the beginning of the pandemic, the behavior of the virus was unknown, and it was therefore legitimate to delay chemotherapy. However, now, in light of current knowledge, it is not. Additionally, lack of knowledge is not an excuse, because health practice essentially involves continuous training and study;
(b) Justice: understood as providing each person with what they are entitled or deserve, corresponding in equity or merit. Delaying or stopping chemotherapy which has been shown to be beneficial because of the pandemic only violates this principle. With justice, these patients must receive their treatment in a timely manner, without delay or suspension;
(c) Beneficence: understood as any action that seeks to do good. If effective therapy is suspended or delayed without justifiable scientific evidence (as we have previously shown), we violate this principle by omission without justification. Every bioethical decision must be supported by the evidence. Additionally, the evidence does not support suspending or delaying treatment which has a clinical benefit;
(d) Autonomy: related to the capacity of each person to decide freely. Any true choice must be based on solid information. Therefore, the oncologist must explain to the patient the benefits and risks of undergoing chemotherapy during this pandemic. The objective will be to listen to the patient, clarify their doubts, and dispel the unfounded myths regarding oncology, chemotherapy, and this infection. Only then will the patient be able to decide freely.

### 2.2. Second Bullet

Regarding the second question, we present the following analysis:

Second item

We will address two ethical views:

(a) Utilitarian ethics: in public health, the R_0_ (basic reproductive ratio) measures the speed with which an infection spreads in a given population (R_0_ = transmission rate/recovery rate) [11]. Additionally, in order to control this pandemic, it is necessary to reduce the effective R_0_, through herd immunity, by vaccinating at least 60% to 70% of the population. In fact, in view of the increased transmissibility of the infection, it may be necessary to vaccinate 80% to 85% of the population.

Utilitarianism is a moral philosophy that states that all action should seek the maximum welfare for the maximum number of people [12]. Thus, it would be lawful to vaccinate the largest number of people (including cancer patients) in order to achieve the greatest good (control the pandemic) for the largest number of people (the general population).

We believe that a utilitarian analysis is not sufficient to determine whether or not it is ethical to vaccinate cancer patients without supporting scientific evidence. These patients have a weakened immune system (due to the neoplastic disease itself and the treatments they receive such as chemotherapy and radiotherapy); their immune response to vaccines may be lower than that of the general population. Thus, we do not know to what extent the vaccination will be effective in cancer patients and will therefore contribute to reducing the spread of the infection.

(b) Double-effect principle: known as a principle of practical reasoning that helps determine the lawfulness of an action that produces (or can produce) two effects, one good and the other bad [13].

We will now analyze the conditions that allow us to apply this principle to our particular case:

(1) The purpose of the act to be pursued must be good: the action is the vaccination of the cancer population and its objective is to prevent symptomatic infection (beneficial objective);
(2) The intention of one who acts must be good and excludes (does not wish, but tolerates) the bad effect that will result from the action: without a doubt, the intention of the one who indicates and the one who proceeds to vaccinate is beneficial.

Adverse effects of vaccines (which should be tolerated) in the general population are usually mild to moderate, local and transient. In cancer patients, they are not yet known. However, messenger RNA vaccines and Ad26.COV2.SCOVID-19 vaccine have no risk of producing COVID-19 infection and no hyperinflammatory response syndromes have been observed in immune-deficient human models;

(3) The action itself must be good or indifferent: a systemic review and meta-analysis (*n* = 17 observational studies including 3268 cases) showed that cancer patients have a higher risk of dying from this viral infection (risk of 24.8 per 100 inhabitants versus 2.2 per 100 inhabitants in the general population). Within this group, the male sex, age over 65 years, comorbidities (especially high blood pressure and chronic obstructive pulmonary disease) and respiratory symptoms (dyspnea, cough and sputum) are described as risk factors for mortality [14]. Furthermore, it is also known that severe or critical COVID-19 events are more frequent in cancer patients than in the general population. Thus, the action of vaccinating is essentially good because it seeks to prevent severe morbidity and increased mortality from COVID-19 infection in this higher-risk population;
(4) There must be a proportionally serious reason to accept the act, i.e., the benefit that is expected to be obtained from the action must be much greater than the damage that the action may produce.

Currently, there is no evidence in scientific studies on the real efficacy of messenger RNA vaccines and Ad26.COV2.SCOVID-19 vaccine in cancer patients. However, in the mRNA-1273 vaccine study, the subgroup with diabetes showed similar efficacy to the general population [7]. We know that diabetic patients have a dysfunctional immune system [15]. Therefore, it can be inferred that benefit will also be found in the oncological subgroup (which, by definition, has a weakened immune system).

The adverse effects of these vaccines in cancer cases are also unknown. However, in a retrospective study (*n* = 370 cases), inactivated influenza virus vaccine administered to cancer patients treated with immunotherapy did not increase new-onset immune-related adverse events [16]. By extrapolation, it can be inferred that cancer patients will not have much greater or more serious adverse events with anti-COVID-19 vaccines than the general population.

Thus, in cancer patients, the potential benefits of vaccination far outweigh the potential adverse events;

(5) Absence of better actions that cause less damage: at present, neither hygienic measures nor physical isolation have been able to stop the advance of the pandemic. Physical isolation is not innocuous. The psychological/social damage to individuals families and the deterioration of the economy is significant. Therefore, there is no better alternative than vaccination.

There is certain controversy about the validity of the double-effect doctrine in the clinical ethics context. Ezio Di Nucci presented eight classic arguments against this principle [17]. On the other hand, Jean Pierre Gury defended this ethical reasoning based on the principle of non-imputability of the direct agent in which one of the two effects (the evil one) is not directly intended [18]. Warren Quinn also defended this principle, pointing out that the difference between direct and indirect harmful agency is what underlies the moral significance of the difference between intended and merely predicted harms [19]. He thinks that “some cases of harming that the doctrine intuitively speaks against are arguably not cases of intentional harming, precisely because neither the harm itself (nor anything itself causally very close to it) is intended” [20].Lawrence Masek also defended the principle of double-effect, based on the so-called strict definition of intention. The author argues that the moral permissibility of an action depends at least in part on how it forms the agent’s character [21].

Regarding our particular case, the main criticisms of the double-effect principle are based on the possible absence of simultaneity of both effects, the consequentialism [22,23], and the problem of proximity [24]. Let us analyze these potential issuespoint-by-point:

(1) Due to it being un ethical to use an evil means to later obtain a good effect, the simultaneity of both effects resulting from the same action is a crucial point to be clarified. In our particular case, the vaccination of cancer patients has a beneficial effect (the potential decrease in morbidity and mortality from COVID-19) and a malefic effect (the potential adverse effect in this population), which are both simultaneous. The beneficial effect is not a consequence of the malefic effect;
(2) Consequentialism states that the moral value of an action depends exclusively on its consequence [22,23]. However, we think every action has an intrinsic moral value [25]. This is certainly true for the case of vaccinating cancer patients;
(3) The problem of proximity describes the evil result that the action attempts and the anticipated negative consequences as indistinguishable. However, the motivation of the agent of the action has no intention of causing an evil effect, but anticipates it. It is only tolerated if the good obtained is proportionally greater than the evil that maybe produced.

On the other hand, the main strengths of the double-effect principle applied to our particular case are the following:

(1) The determination of the proportionality relationship between the beneficial effect and the malefic effect;
(2) This principle considers the absence of alternatives actions that cause less damage.

These strengths support the double-effect principle as a valid ethical reasoning that favors decision-making on the vaccination of cancer patients during this pandemic.

## 3. Discussion

Is it ethical to postpone or suspend cancer treatments which offer a statistically significant benefit in the quality of life and survival in cancer patients during this pandemic? Despite the pandemic, based on the best available evidence, it is unethical to postpone or suspend adjuvant, neoadjuvant, or palliative chemotherapy treatments (or immunological therapy, hormonal therapy, radiotherapy, and target therapies) which have demonstrated a statistically significant benefit in terms of the quality of life and survival in cancer patients. However, the bioethics are based on virtue. Additionally, the virtue of prudence is very important. The fact that a patient regularly attends a chemotherapy center increases the risk of contagion during this pandemic. Additionally, the mortality rate is higher in infected cancer patients. Therefore, all necessary precautions must be taken to reduce a patient’s risk of contagion when attending a healthcare center. Measures such as the personalized transport of patients, the disinfection of all surfaces in transport vehicles and the chemotherapy room, the use of masks and their frequent replacement, maintaining a distance of at least 1.5 m, frequent hand washing, etc., should all be considered.

Should we vaccinate cancer patients against COVID-19 if scientific studies have not included this subgroup of patients? In conclusion, considering the principle of double-effect, it is lawful and ethical to vaccinate the cancer population. The autonomy and decision of the patient should be always respected (who has been previously well-informed by their treating oncologist).

Now, we point out three relevant facts:

(1) The efficacy of messenger RNA vaccines and Ad26.COV2.SCOVID-19 vaccine in cancer patients is not yet known;
(2) We do not know the percentage of protection from asymptomatic infection in the general population;
(3) The vaccines developed against the original virus have been found to be less effective against variant viral B.1.351 (this variant contains the E484K mutation that has caused so much concern in B.1.351. This mutation is thought to allow the virus to escape from some of the body’s immune response) [26].

Therefore, maintaining hygiene and distancing should continue to be respected for the time being.

Finally, we mention that one limitation of the present article corresponds to the absence of other relevant ethical dilemmas in the context of comprehensive cancer treatment in times of the COVID-19 pandemic. We leave this task for future work.

## Data Availability

Not applicable.
